# Effectiveness of Shrinkage and Variable Selection Methods for the Prediction of Complex Human Traits using Data from Distantly Related Individuals

**DOI:** 10.1111/ahg.12099

**Published:** 2015-01-20

**Authors:** Swetlana Berger, Paulino Pérez‐Rodríguez, Yogasudha Veturi, Henner Simianer, Gustavo de los Campos

**Affiliations:** ^1^ Animal Breeding and Genetics Group, Department of Animal SciencesGeorg‐August‐University GoettingenAlbrecht‐Thaer‐Weg 3GoettingenGermany; ^2^ Colegio de PostgraduadosCarretera México‐Texcoco Km. 36.5, MontecilloTexcocoEstado de MéxicoMéxico; ^3^ Department of Biostatistics, University of Alabama at Birmingham, RPHB 317CRyals School of Public HealthBirminghamALUSA

**Keywords:** Whole genome regression, prediction accuracy, variable selection, shrinkage estimation, linkage disequilibrium, genetic architecture, missing heritability

## Abstract

Genome‐wide association studies (GWAS) have detected large numbers of variants associated with complex human traits and diseases. However, the proportion of variance explained by GWAS‐significant single nucleotide polymorphisms has been usually small. This brought interest in the use of whole‐genome regression (WGR) methods. However, there has been limited research on the factors that affect prediction accuracy (PA) of WGRs when applied to human data of distantly related individuals. Here, we examine, using real human genotypes and simulated phenotypes, how trait complexity, marker‐quantitative trait loci (QTL) linkage disequilibrium (LD), and the model used affect the performance of WGRs. Our results indicated that the estimated rate of missing heritability is dependent on the extent of marker‐QTL LD. However, this parameter was not greatly affected by trait complexity. Regarding PA our results indicated that: (a) under perfect marker‐QTL LD WGR can achieve moderately high prediction accuracy, and with simple genetic architectures variable selection methods outperform shrinkage procedures and (b) under imperfect marker‐QTL LD, variable selection methods can achieved reasonably good PA with simple or moderately complex genetic architectures; however, the PA of these methods deteriorated as trait complexity increases and with highly complex traits variable selection and shrinkage methods both performed poorly. This was confirmed with an analysis of human height.

## Introduction

The availability of genomic data has revolutionised the statistical analysis of human diseases and traits. The development of methods that can accurately predict the genetic risk associated with these diseases and complex human traits can have a great impact on public health (e.g., Guttmacher et al., 2002; Simon‐Sanchez et al., 2009). Modern genotyping and sequencing technologies can deliver massive amounts of information about the human genome, which are necessary for the prediction of genetic risk. However, the incorporation of genomic data into prediction remains challenging.

In recent years, a large number of genome‐wide association studies (GWAS) have been conducted (e.g. http://www.genome.gov/gwastudies/). These studies have identified unprecedented numbers of variants associated with important complex traits and diseases. In some cases, the variants identified so far explain a sizable proportion of the variance of the trait or disease. Examples of these include Crohn's disease, age‐related macular degeneration and Type I diabetes (Manolio et al., 2008; Goldstein, 2009). However, for the great majority of traits and diseases, the variance accounted for by GWAS hits is small, regardless of whether they are moderately or highly heritable (Allen et al., 2010). Consequently, the use of genomic information for prediction of risk for diseases with complex genetic architectures remains limited. This problem, the so‐called “missing heritability” of complex traits, has been discussed extensively by multiple authors (e.g., Maher, 2008; Manolio et al., 2009; Eichler et al., 2010).

Although several factors contribute to the “missing heritability” problem, a major explanation resides in the lack of power of standard GWAS to detect small‐effect variants. Recent studies have shown that prediction accuracy can be improved by including in risk scores information of allele content at variants that show suggestive, albeit not statistically significant, association with the trait or disease being studied (Allen et al., 2010). However, most risk score methods are still based on a limited number of loci and alleles at different loci that are either equally weighted or weighted using statistics derived from single‐marker‐based association tests. Several authors (de los Campos et al., 2010; Yang et al., 2010; Makowsky et al., 2011; Speed et al., 2012) have suggested that a potentially better approach may consist of regressing phenotypes on whole‐genome markers simultaneously using a whole‐genome regression (WGR) approach like the one originally proposed by Meuwissen et al. (2001).

WGR has been used with human data for estimation of the proportion of variance that can be explained by regression of phenotype on markers (e.g., Yang et al., 2010; Speed et al., 2012) and for the assessment of prediction accuracy (e.g., Makowsky et al., 2011; de los Campos et al., 2013b). Using a genomic best linear unbiased predictor (GBLUP) model and data from distantly related individuals, Yang et al. (2010) showed that simultaneous regression on a large set of ∼300,000 common single nucleotide polymorphisms (SNPs) could explain roughly 50% of the heritability of human height. This encouraging result suggested that a large fraction of the missing heritability could be recovered by using regression methods based on large panels of whole‐genome markers.

Accuracy of prediction of yet‐to‐be observed phenotypic or disease outcomes is arguably one of the most important features of a model when it comes to potential use of the method for precision medicine. It is well established that prediction accuracy of WGR methods is highly affected by genetic relationships (e.g., Makowsky et al., 2011) and it is not clear whether WGR methods that have been proved accurate for prediction of complex traits with family data (VanRaden et al., 2009; Crossa et al., 2010; Makowsky et al., 2011) will also be effective when applied to distantly related individuals, which are often of interest in human genetic applications.

According to Goddard (Goddard & Hayes, 2009), when WGR is applied to distantly related individuals, the prediction accuracy depends on two main factors: (1) the proportion of variance that can be explained by regression on the marker set (this depends largely on the extent of linkage disequilibrium (LD) between alleles at the markers and those at causal loci and, according to Yang et al. (2010) could be estimated using variance components), and (2) the accuracy of estimates of marker effects. These are two opposing forces: as we add more markers in the prediction equation the proportion of variance explained by markers potentially increases; however, more marker effects need to be estimated and the individual accuracy of estimates of effects will typically decrease. Therefore, in finite samples is not exactly clear that methods that have a higher proportion of variance explained in the training data will also be best for prediction of yet‐to‐be‐observed outcomes. For example, in a recent study on prediction of human height using GBLUP, de los Campos et al. (2013b) showed that, with distantly related individuals, prediction accuracy increased as markers were added to the model up to a saturation point beyond which it decreased. This result suggests that the analysis and prediction of complex traits may benefit from the use of models that combine variable selection and shrinkage within a single framework.

In the last two decades, important developments in the area of penalised and Bayesian estimation procedures have led to a number of methods for implementing *large‐p‐small‐n* regressions, including various methods that combine shrinkage estimation and variable selection. An overview of different penalised methods can be found in Hastie et al. (2005) and an overview of Bayesian methods for variable selection and shrinkage estimation (with a focus on genetic applications) is given by Gianola (2013) and de los Campos et al. (2013a). In animal and plant breeding, use of these methods has led to a substantial improvement in prediction accuracy (Habier et al., 2011; Heslot et al., 2012). Several studies have compared shrinkage and variable selection methods from a predictive perspective in animal and plant breeding applications (e.g., Habier et al., 2007; Calus et al., 2008; Verbyla et al., 2009; Daetwyler et al., 2010; Gao et al., 2013; Wimmer et al., 2013). Simulation studies have suggested superiority of variable selection methods over shrinkage estimation procedures. However, real data have not always confirmed that (de los Campos et al., 2013a) and in empirical analyses the predictive performance of different regression methods has been very similar, perhaps reflecting the fact that the architecture of most traits is more complex than often assumed in simulation studies. Most of the studies in plant and animal breeding are based on family data. The few studies (e.g., Habier et al., 2007; Gao et al., 2013 in breeding populations and Makowsky et al., 2011 or de los Campos et al., 2013a with human data) that have assessed prediction accuracy with distant relatives have found that the prediction accuracy of WGRs models deteriorates quickly as the genetic distance between training and testing populations increases. In principle, variable selection methods are better suited to detect variants that are in strong LD with QTL, and this should make these methods more robust with respect to the effects of genetic distance on prediction accuracy (e.g., Habier et al., 2007).

However, the performance of these methods for prediction with human data so far has not been studied in detail. Indeed, in applications involving human data, most of the studies (Yang et al., 2010; Makowsky et al., 2011; de los Campos et al., 2013b) have used ridge‐regression type estimators that do not involve variable selection or differential shrinkage of estimated effects. Zhou et al. (2013) used WGR models that combine variable selection and shrinkage using data from distantly related individuals; unfortunately, the study did not evaluate the prediction accuracy. Importantly, the factors that affect prediction accuracy in the analysis of family data can be different than those that affect prediction accuracy when training and validation samples are distantly related. Indeed, with family data, co‐segregation of alleles at markers and at quantitative trait loci (QTL) plays a major role, and can induce linkage between markers and QTL at distant positions. Under these conditions, variable selection is difficult to perform and may not be needed because signals generated by QTL can be tracked by markers that are far apart from a QTL. This type of linkage is not present when training and validation samples are distantly related, and we lack research about the relative effectiveness of shrinkage and variable selection methods with data from distantly related individuals.

Therefore, the main goal of this study was to assess the predictive performance of different types of WGR methods, including both shrinkage estimation procedures and methods that perform variable selection, when used for prediction of complex traits and with distantly related individuals. We considered three statistical methods that differ in the prior distribution of marker effects and consequently yield different types of estimates. First, a model with Gaussian distribution of marker effects (the GBLUP) was used; this ridge‐regression‐type method induces homogeneous shrinkage of marker effects. Second, a *scaled‐t* prior for marker effects (labelled as Bayes A by Meuwissen et al., 2001) was used; a method that induces an effect‐size dependent shrinkage of estimates (Gianola, 2013). Finally, a Spike‐Slab model (e.g., George & McCulloch, 1993; Ishwaran & Rao, 2005) was used, which combines variable selection and shrinkage. Recent methodological developments introduced by Zhou et al. (2013) allow implementation of a Spike‐Slab model even with a very large numbers of markers.

The performance of these methods was assessed with simulated and real data. Our simulation comprised different scenarios pertaining to the complexity of the trait (in terms of number of large‐effect loci) and the pattern of LD between markers and causal or QTL. The results obtained from simulation studies were validated by analysis of human height measured on distantly related individuals.

## Materials and Methods

In the classical quantitative genetic model, a continuous trait $y_{i}$ is described as a sum of three components: the population mean (μ), a random component reflecting the genetic factors, the so‐called genetic value $u_{i}$, and a random model residual (${ɛ}_{i}$) usually assumed to be identically and independently normal distributed with zero mean and variance $\sigma_{ɛ}^{2}$.

In genomic models, the genomic values $u_{i}$ are approximated using regressions on marker genotypes. For instance, in an additive model one can set $u_{i}=\sum_{j=1}^{p}X_{ij}\beta_{j}$, where $X_{ij}\in \left\{0,1,2\right\}$ represents the allele dosage at the *j* ^th^ locus of the *i* ^th^ individual and $\beta_{j}$ represents the corresponding marker effect. Thus, the model for *p* markers can be expressed as:

(1) $$y_{i}=\mu +\sum_{j=1}^{p}X_{ij}\beta_{j}+{ɛ}_{i},i=1,...,n.$$

In WGR methods, the number of effects to be estimated can vastly exceed the number of data points (i.e., p≫n). Thus, the estimation of effects in the model described above requires the use of some type of regularised regression procedure such as penalised or Bayesian regression. In Bayesian regressions, the type and extent of shrinkage of estimates of effects is controlled by the choice of prior for marker effects.

To cover a wide range of methods, in this study we considered two extreme approaches (GBLUP a shrinkage estimation procedure and the Spike‐Slab, a method that combines variable selection and shrinkage) and an intermediate one (Bayes A) that induce differential shrinkage of estimates of effects.

The GBLUP model is obtained by assigning independent identically distributed (IID) normal before the marker effects, that is: $\beta_{j}∼N\left(0,\sigma_{\beta }^{2}\right),\;\;j=1,...,p$. This approach yields estimates equivalent to those from ridge regression, where all effects are shrunk toward zero to a similar extent. Using the expectation of *i*th phenotype $y_{i}$ (given the genotypes and marker effects), and the genomic value $u_{i}=\sum_{j=1}^{p}X_{ij}\beta_{j}$, we rewrite equation (1) as $y_{i}=u_{i}+{ɛ}_{i},i=1,...,n$. Thus, the genomic value is also normal: $u∼N(0,\sigma_{u}^{2}G)$ with a genomic relationship matrix, which is obtained as a cross product of genotype readings $G=\left\{G_{ik}\right\}=\frac{1}{\sum_{j}2p_{j}\left(1-p_{j}\right)}{\mathrm{XX}}^{\prime }$ [$p_{j}$ is the minor allele frequency (MAF) at the *j*th locus] and a genomic variance component $\sigma_{u}^{2}=\sum_{j=1}^{p}2p_{j}\left(1-p_{j}\right)\sigma_{\beta }^{2}$. Therefore, the GBLUP could be implemented in Bayesian settings as a random effect model with a variance–covariance structure represented by $\sigma_{u}^{2}G+\sigma_{ɛ}^{2}I$, assuming for example a scaled inverse χ^2^ density as a prior distribution for variance components $\sigma_{u}^{2}$ and $\sigma_{ɛ}^{2}$.

Above we described the GBLUP model that one obtains by regressing phenotypes on markers using IID normal priors for marker effects. This model can be fitted by either regressing phenotypes on markers explicitly, or using an equivalent model based on a genomic relationship matrix $G\propto {\mathrm{XX}}^{\prime }$. Some authors (Speed et al., 2012) have proposed alternative ways of computing genomic relationships that account for LD; therefore, we also fitted the GBLUP model applying the method proposed by (Speed et al., 2012) to compute **G** using the LDAK software (available at http://dougspeed.com/); we refer to this method as to GBLUP‐ldak.

In Bayes A, markers are assumed to follow IID scaled‐*t* densities (an example for *t*‐scaled prior with 5 degrees of freedom is given in Fig. S1). In practice it is convenient to represent this density as an infinite mixture of scaled‐normal densities: $t\left(\beta_{j}|\mathrm{df},S\right)=\int N(\beta_{j}|0,\sigma_{\beta_{j}}^{2})\chi^{-2}\left(\sigma_{\beta_{j}}^{2}|\mathrm{df},S\right)\partial \sigma_{\beta_{j}}^{2}$, where $N(\beta_{j}|0,\sigma_{\beta_{j}}^{2})$ is a normal density with null mean and variance $\sigma_{\beta_{j}}^{2}$ and $\chi^{-2}\left(\sigma_{\beta_{j}}^{2}|\mathrm{df},S\right)$ is a scaled‐inverse χ^2^ density with degree of freedom *df* and scale parameter *S* (e.g., Gianola et al., 2009).

In the Spike‐Slab model, the prior assigned to marker effects is a mixture of two distributions: one (the spike) with small variance concentrated around zero that corresponds to small or no effects and the other (the slab) is a flat distribution with large variance that is linked to large marker effects. The spike can be represented by a continuous distribution centred at zero and with very small variance or by a point mass at zero. We concentrate on the prior introduced by George and McCulloch (1993), a mixture of two normal distributions. Conditional on the proportion of large effects, π, and on variance parameters, the distribution of marker effects is given by $p\left(\beta_{j}|\pi ,\sigma_{\beta_{1}}^{2},\sigma_{\beta_{2}}^{2}\right)=\pi N\left(\beta_{j}|0,\sigma_{\beta_{1}}^{2}\right)+\left(1-\pi \right)N\left(\beta_{j}|0,\sigma_{\beta_{2}}^{2}\right)$, where $\sigma_{\beta_{1}}^{2}$ reflects the variability in large effects and $\sigma_{\beta_{2}}^{2}$ is the variance component of small effects. An example for π=0.15 is represented in Figure S1.

Recently, Zhou et al. (2013) proposed an efficient method to implement the Spike‐Slab model. In their approach, called Bayesian sparse linear mixed model (BSLMM), they represent marker effects as the sum of two components: small effects $\alpha_{j}∼N\left(\alpha_{j}|0,\sigma_{\alpha }^{2}\right)\;$, assigned to all markers and sparse effects $\gamma_{j}∼\pi N\left(\gamma_{j}|0,\sigma_{\gamma }^{2}\right)+\left(1-\pi \right)\delta_{0}$ (a mixture of a normal and a point‐mass‐at‐zero distribution), which are assigned to a proportion of markers π, so that the total effect of the *j* ^th^ SNP $\beta_{j}=\alpha_{j}+\gamma_{j}$ is a mixture of normal distributions $\pi N\left(\beta_{j}|0,\sigma_{\alpha }^{2}+\sigma_{\gamma }^{2}\right)+\left(1-\pi \right)N\left(\beta_{j}|0,\sigma_{\alpha }^{2}\right)$. Zhou et al. (2013) specified this model using a re‐parameterization which greatly facilitates computations.

All simulations as well as subsequent statistical analyses of simulated and real data were implemented in R (R Core Team, 2014). In this study, the GBLUP and Bayes A methods were fitted using the Gibbs Sampler algorithm implemented in the R package, BGLR (Pérez and de los Campos, 2014). The Spike‐Slab model was fitted using the BSLMM method, which is included in the GEMMA software package (http://stephenslab.uchicago.edu/software.html).

## Simulation and Real Data Analysis

### Data

The genotypes used for simulation and in the real data analysis came from by NIH‐funded gene–environment association studies (GENEVA, http://www.genevastudy.org), which is a consortium of 16 genome wide association studies. We used a subset of GENEVA consisting of data from the Nurses’ Health Study and the Health Professionals’ Follow‐up Study studies. Samples were genotyped using the Affymetrix Genome‐Wide Human SNP Array 6.0 with about 780 K SNPs. The GENEVA data set contains phenotypic and genotypic records of *n* = 5,961 individuals (3,391 women and 2,570 men) with average age of 57.2 years (SD = 7.7 years) and average height 170.2 cm (SD = 9.6 cm). For the real data analysis we used adult height (adjusted for age, sex and affiliation to case or control group) as the phenotype.

### Quality Control Procedures

We removed all markers with proportion of missing genotypes per SNP ⩾0.01 and all individuals with a proportion of missing genotypes per individual ⩾0.05. Furthermore, on the basis of the available pedigree information, we also removed all nominally related individuals and individuals with a Hispanic genomic background such that only individuals of Caucasian origin remained in the data set. We also set a lower threshold of 0.01 for MAF, so that after quality control of the genomic data sample size was 5,758 individuals and 673,197 SNPs loci remained.

### Simulation

We aimed at investigating the performance of three models, which apply different types of shrinkage of effect estimates, under different genetic architectures and varying levels of LD between markers and QTL. The simulation was conducted using true genotypes (see details above) and simulated phenotypes.

#### Markers and QTL

SNPs were randomly divided into two subsets: 350K SNPs were designated as markers and the rest (∼323K) were used as a pool for sampling subsets of QTL (5K, in each replicate). The 5K QTL were sampled from the pool of 323K loci either completely at random (RAND) or by oversampling among the loci with low minor allele frequency (LOW‐MAF). In this case sampling probabilities were set to target 75% of the QTL with MAF < 0.05, 25% of the QTL with MAF between 0.05 and 0.15, no QTL had a MAF > 0.15. In the LOW‐MAF scenario, the distributions of allele frequencies at markers and at QTL were expected to be different, and this was expected to influence the extent of LD between markers and QTL. Therefore, for each replicate, we used PLINK (Purcell et al., 2007) to compute the pairwise squared correlation *r* ^2^ between genotypes at the QTL and those at the two flanking markers.

#### Genetic architecture

We assumed that only a subset of QTL had large effects, whereas the rest of them had small effects. We considered three different scenarios: in the first one all QTL effects were sampled from IID normal densities $N(\beta_{j}|0,\sigma_{\beta }^{2})$. In the second and third scenarios we randomly chose *p* = 50 or 250 SNPs, respectively, and sampled their effects from a normal density with a large (see next) variance, the rest of the QTL effects were sampled from a normal density with a smaller variance. We set the variance parameters of the two normal densities used to sample effects in scenarios 2 and 3 to target a heritability (*h* ^2^) of 0.5 and a partition of the genetic variance (hereinafter called *pve*) where large effect QTL explain either 25% or 75% of genetic variance in scenarios 2 and 3.

#### Simulation of phenotypes

The phenotypes were constructed according to an additive model $y_{i}=\sum_{j=1}^{5000}Z_{ij}\beta_{j}+{ɛ}_{i}$ for i=1,...,n, where model error ${ɛ}_{i}$ and marker effects $\beta_{j}$ follow normal distributions with zero mean and $Z_{ij}$ are the genotype readings at causal loci. The variance of the residual term $V({ɛ}_{i})=0.5$ was kept fixed across all scenarios, whereas the variance of marker effects $V(\beta_{j})$ varied from scenario to scenario, depending on the number of large effect QTL, amount of genetic variance explained by these large effects QTL, and the distribution of MAFs in QTL.

### Data Analyses

We analysed the simulated data using markers, QTL or markers and QTL. The first scenario involved imperfect LD between markers and QTL, the last two contained the causal variants in the panel and therefore were perfect LD scenarios.

#### Genomic heritability

For the GBLUP, the estimated genomic heritability $h_{G}^{2}=\frac{\sigma_{g}^{2}}{\sigma_{g}^{2}+\sigma_{ɛ}^{2}}$ was defined as the ratio between the variance explained by genomic factors, $\sigma_{g}^{2}$, and the phenotypic variance, $\sigma_{p}^{2}=\sigma_{g}^{2}+\sigma_{ɛ}^{2}$; in the G‐BLUP, $h_{G}^{2}$ was estimated based on posterior samples collected using the BGLR‐package.

For Bayes A, the BGLR‐package did not provide the estimates of genomic heritability directly. In this model, a scaled‐inverse χ^2^ distribution is assigned to the variance of the effects $\beta_{j}$. Therefore, we have $E\left(\sigma_{\beta }^{2}\right)=\frac{S_{0}}{\mathrm{df}-2}$; using this we can define the genomic variance as follows: $\sigma_{g}^{2}=\sum_{j=1}^{p}2p_{j}\left(1-p_{j}\right)\frac{S_{0}}{\mathrm{df}-2}$, where $p_{j}$ stands for allele frequency at locus *j*. With this, the genomic heritability can be defined as $h_{G}^{2}=\frac{\sum_{j=1}^{p}2p_{j}\left(1-p_{j}\right)\frac{S_{0}}{\mathrm{df}-2}}{\sum_{j=1}^{p}2p_{j}\left(1-p_{j}\right)\frac{S_{0}}{\mathrm{df}-2}+\sigma_{ɛ}^{2}}$. We also estimated this parameter using posterior samples collected using the BGLR‐package.

GEMMA provided posterior samples of $\mathrm{PVE}\left(\beta ,u,\tau^{-1}\right)=\frac{V(X\beta +u)}{V\left(X\beta +u\right)+\tau^{-1}}$ (Zhou et al., 2013), which describes total proportion of variance in phenotype explained by the sum of the “sparse” (Xβ) and random effect (**u**). Essentially. this quantity meets definition of genomic heritability, we used posterior mean of PVE to obtain the estimate of genomic heritability. In addition to estimates of genomic heritability, we report the *R* ^2^ between phenotypes and predictions in the training data set as a measure of goodness of fit. This was only done for the GBLUP and Bayes A because GEMMA does not provide predictions for the training data set.

#### Assessment of prediction accuracy

To assess prediction accuracy, in both the simulated and real data, we replicated 30 times a training–testing (TRN–TST) validation design (Hastie et al., 2005). In each TRN–TST experiment, data were randomly split into two disjoint sets, 5258 data points in the TRN and from the remaining 500 individuals, we retained for validation only the ones whose genomic pairwise relationships with individuals in the TRN group did not exceed 1/8; these were typically ∼400 individuals. In the analysis of real phenotype (adjusted human height), we used the same subset of SNPs that were used in the “only marker” scenario in simulation studies and the same mapping of individuals to TRN/TST groups. We assessed prediction accuracy using the Pearson's product–moment correlation between the true and predicted phenotypes $\mathrm{cor}(y,\hat{y})$ in the validation set.

## Results

### Results from Simulation Studies

The empirical quantiles of the distribution of MAF at different sets of loci are given in Table 1. In the RAND scenario, the empirical distribution of the MAF at QTL and markers were very similar; this was expected because both sets of loci were sampled at random. However, as intended, the empirical distribution of MAF at QTL in the LOW‐MAF scenario had, relative to the same distribution at the marker loci, an over representation of loci in the low MAF spectra.

**Table 1:** Empirical percentiles of the distribution of minor allele frequency for markers and for QTL in simulated data in both sampling scenarios

	Percentiles of the distribution of minor				
	allele frequency				
Set (scenario)	5%	10%	25%	50%	95%
Markers	0.0298	0.0498	0.1115	0.2268	0.4713
QTL (RAND)	0.0302	0.0501	0.1117	0.2273	0.4713
QTL (LOW‐MAF)	0.0133	0.0169	0.0279	0.0461	0.1383

The 5%, 10%, 25%, 50% and 95% percentiles for marker data set and for QTL in both sampling scenarios, averaged over 30 replicates.

LD is allele‐frequency dependent; therefore, based on results of Table 1 one would expect that the extent of Marker‐QTL LD will vary between scenarios. Table 2 provides a summary of estimates of LD between QTL and the two flanking markers by scenario. The average of *r* ^2^ over 30 Monte‐Carlo (MC) replicates in the RAND‐scenario was 0.624 with a standard deviation (SD) of 0.286. However, the average of pairwise. *r* ^2^ in the LOW‐MAF‐scenario was three times smaller.

**Table 2:** Summary statistics of pairwise LD measure in simulated data in both sampling scenarios

		Quantiles				
Scenario	Average *r* ^2^ (SD)	5%	25%	50%	75%	95%
RAND	0.624 (0.286)	0.223	0.344	0.609	0.941	0.996
LOW‐MAF	0.206 (0.333)	0.001	0.007	0.029	0.203	0.982

Summary statistics of pairwise LD, measured as squared correlation *r* ^2^ between the QTL and markers, flanking markers on either side in the RAND and LOW‐MAF scenarios; *r* ^2^ is averaged over 30 Monte‐Carlo replicates, with standard deviation given in parentheses and 5%, 25%, 50%, 75% and 95% quantiles.

### Estimated Genomic Heritability and Goodness of Fit

**Figure 1:**
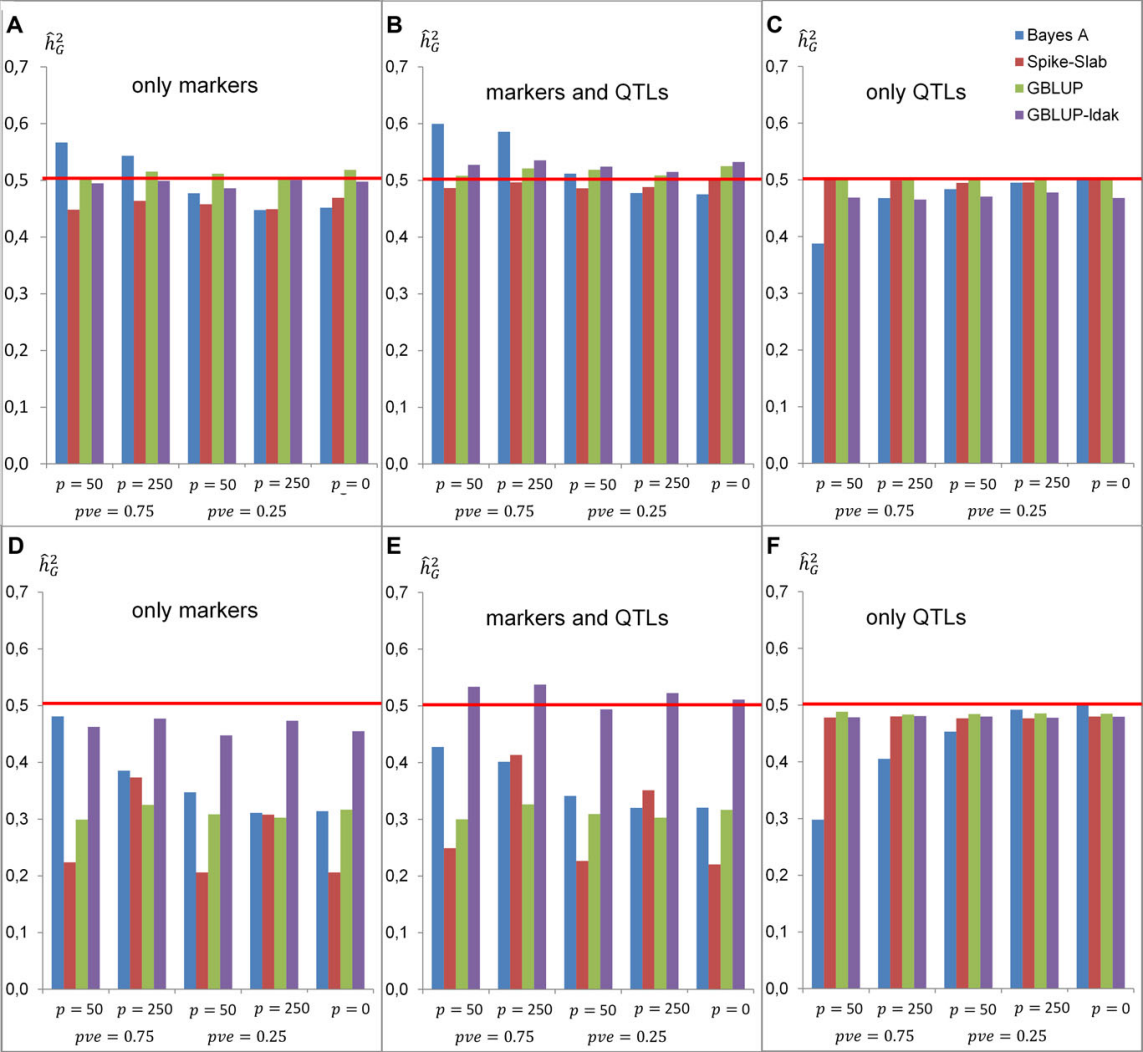
Estimates of genomic heritability. Average (over 30 Monte Carlo replicates) estimates of genomic heritability (vertical axis) by: simulation scenario (RAND upper panel: A–C; LOW‐MAF in lower panel: D–F), genetic architecture (*p* = number of large effect QTL, *pve* = proportion of genetic variance explained by large effect QTL), model (GBLUP, GBLUP‐ldak, Bayes A and Spike‐Slab) and data used (only markers, markers and QTL, or only QTL). Red lines refer to the simulated heritability ${\hat{h}}^{2}=0.5$.

The average (over MC replicates) estimated genomic heritabilities obtained by simulation scenario (RAND in the upper panel, LOW‐MAF in the lower panel), statistical method (Bayes A, Spike‐Slab, GBLUP and GBLUP‐ldak), information used (markers, markers+QTL and QTL) and genetic architecture are shown in Figure 1.

#### QTL‐based analysis

When only QTL genotypes were used to fit models to data simulated with the RAND scenario (Fig. 1C), the GBLUP and Spike‐Slab models gave an average estimate of genomic heritability that was very close to the simulated heritability, suggesting that these two methods have almost no bias with the sample size used in this study. GBLUP‐ldak generally underestimated heritability and Bayes A yielded downwardly biased estimates when the genetic architecture had a few markers explaining a sizable proportion of genetic variance (e.g., *pve* = 0.75, *p* = 50 in Fig. 1C). In the LOW‐MAF scenario (Fig. 1F), GBLUP, Spike‐Slab and GBLUP‐ldak showed almost unbiased estimates, but Bayes A continued to deliver downwardly biased estimates in scenarios where large‐effect QTL explained a sizable fraction of genetic variance (e.g., *pve* = 0.75, *p* = 50 in Fig. 1F).

#### Marker‐based analysis

It is important to note that, due to imperfect marker‐QTL LD when only markers are used in the analysis, the true proportion of variance that can be explained by regression on markers [the so‐called genomic heritability, (e.g., de los Campos et al., 2014)] can be lower than the trait heritability. Therefore, even in simulations, the population value of the genomic heritability is unknown and therefore we can compare results across models but we cannot assess bias. In the RAND scenario the estimates derived with the GBLUP models (see Fig. 1A) were very close to the simulated trait heritability. However, the estimates obtained with the Spike‐Slab model suggested some extent (of the order of 10%) of missing heritability. Bayes A yielded estimates similar to those of the Spike‐Slab with complex genetic architectures but tended to overestimate the genomic heritability with simpler genetic architectures.

In the LOW‐MAF scenario (see Fig. 1D) estimates of genomic heritability varied substantially between methods and genetic architectures: the GBLUP and Bayes A yielded a great extent of missing heritability. In comparison, GBLUP‐ldak yielded a much smaller extent of missing heritability and Spike‐Slab estimated an extent of missing heritability that was small in scenarios in which large effect QTL contributed a sizable proportion of variance and increased—to the point of getting very close to GBLUP—as trait complexity increased.

Finally, as one could expect, the analysis based on markers and QTL (Fig. 1B and E) yielded estimates that were intermediate between the QTL only and marker only cases in the RAND scenario and were very close to the analysis based on markers in the LOW‐MAF scenario.

The *R* ^2^ between true and the predicted phenotypes in the training data sets, averaged over 30 MC replicates, is represented in Figure S2. We do not present results for GEMMA because this software does not provide predictions for the training data set. In the perfect LD scenario (only QTL genotypes used, Fig. S2C and F), the *R* ^2^ was between 60% and 70%, suggesting some overfitting (the simulated heritability was 0.5). The evidence of overfitting increased slightly when markers were used. The clearest sign of overfitting was observed with Bayes A in the LOW‐MAF scenario. In the analysis based on markers only (Fig. S2A and D), the three models behaved very differently: GBLUP showed the lowest *R* ^2^, and this statistic did not vary much between scenarios. However, GBLUP‐ldak showed much higher *R* ^2^ than GBLUP and the value of this goodness of fit statistics for this model was also very stable across simulation scenarios. Finally, Bayes A showed a pattern with higher *R* ^2^ than GBLUP in scenarios involving large‐effect QTL with sizable contribution to additive variance. However, the *R* ^2^ in the training data set of Bayes A decreased as the genetic architecture of the simulated trait became more complex, to a point that the Bayes A approached GBLUP when there were no large effect QTL.

### Prediction Accuracy

**Figure 2:**
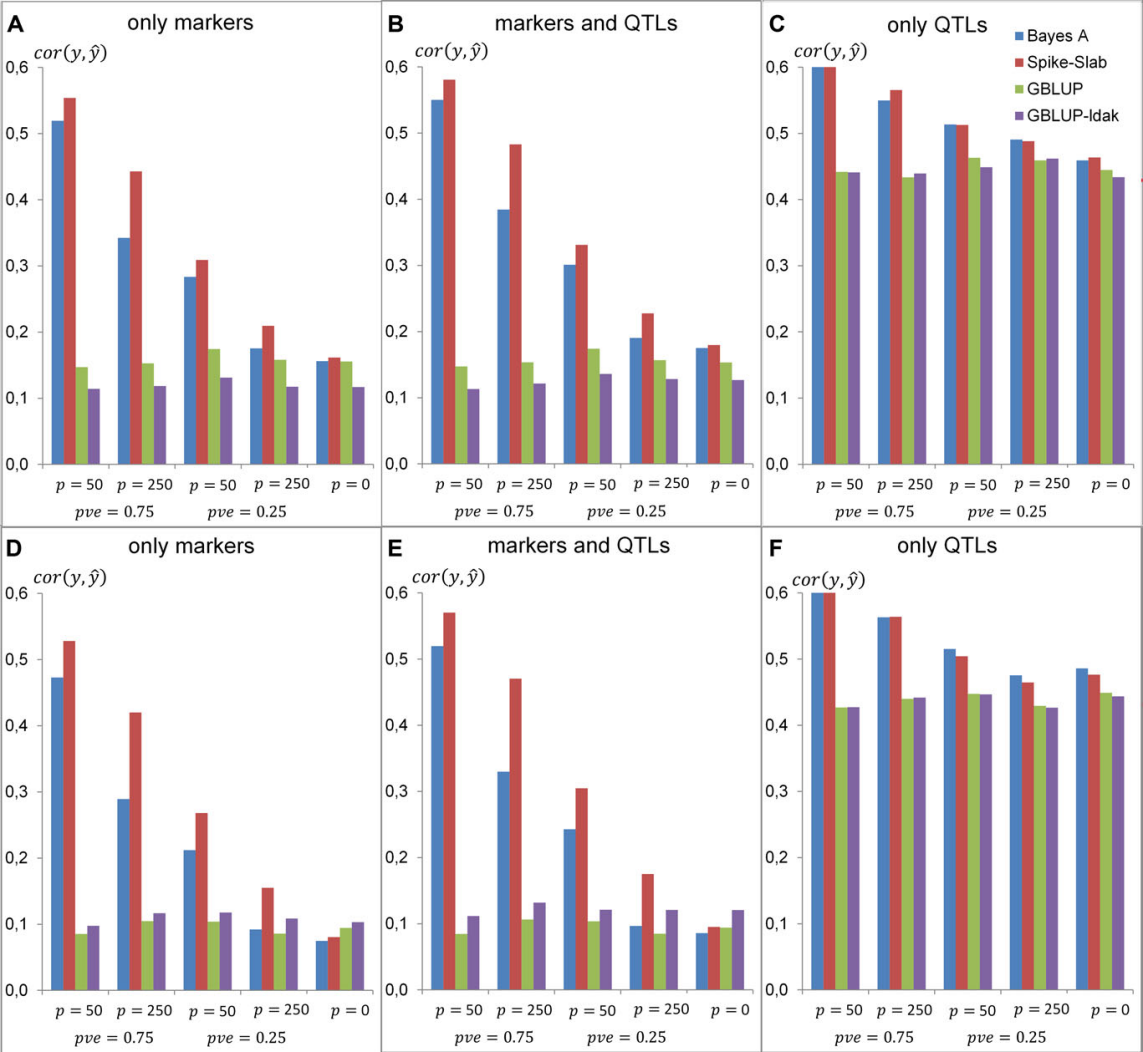
Correlation between phenotypes and genomic predictions in training data sets. Correlation (average over MC replicates) between phenotypes and genomic predictions in training data sets, by: simulation scenario (RAND upper panel: A–C; LOW‐MAF in lower panel: D–F), genetic architecture (*p* = number of large effect QTL, *pve* = proportion of genetic variance explained by large effect QTL) data used (only markers, markers and QTL, or only QTL) and analysis method (GBLUP, GBLUP‐ldak, Bayes A and Spike‐Slab).

Figure 2 displays the correlation (average over 30 MC replicates) between phenotypes and predictions in testing data sets. Plots were sorted by simulation scenario (RAND or LOW‐MAF), genetic architecture (number of large effect‐QTL and proportion of genetic variance explained by large effect QTL), data used (QTL, markers, or markers+QTL) and analysis methods (Bayes A, Spike‐Slab, GBLUP and GBLUP‐ldak).

#### Impacts of LD

The comparison of the prediction accuracy achieved using only QTL (Fig. 2C and F) and those obtained using only markers (Fig. 2A and D) sheds light on the impacts of LD on prediction accuracy. As expected, the maximum prediction accuracy across methods and simulation scenarios was achieved when only QTL genotypes were used for model fitting and prediction (perfect LD scenario). When markers in imperfect LD with QTL were introduced, prediction accuracy was reduced markedly. The adverse effects of imperfect LD between markers and QTL were more marked in the GBLUP and GBLUP‐ldak and less adverse for model Spike‐Slab and Bayes A and in scenarios with simpler genetic architectures; however as the genetic architecture of the trait become more complex, the superiority of these two methods, relative to GBLUP diminished.

#### Statistical method

Overall, GBLUP and GBLUP‐ldak had the worst predictive performance; this was particularly clear when only markers or markers and QTL were used. Bayes A performed considerably better than the GBLUP and the Spike‐Slab performed even better than Bayes A indicating clear benefits of methods inducing differential shrinkage of estimates relative to methods like the GBLUP that induce homogeneous shrinkage of estimates.

#### Genetic architecture

The highest prediction accuracy was obtained in scenarios where a small number of QTL with large effects (*p* = 50) explained a large proportion of the genetic variance (*pve* = 75%). The superiority of the Spike‐Slab or Bayes A over the GBLUP was maximum when the genetic architecture was simple; however, the differences between the prediction accuracy of Bayes A and Spike‐Slab, relative to GBLUP methods diminished as the trait architecture became more complex. Although, the prediction accuracy of the GBLUPs was not greatly affected by the genetic architecture of the trait, in analyses based on markers or markers and QTL, there was a small but systematic trend suggesting that GBLUP outperformed GBLUP‐ldak in the RAND scenario and the opposite was true in the LOW‐MAF scenarios.

For each MC replicate we computed differences in prediction accuracy, measured by differences in correlations $\mathrm{cor}(y,\hat{y})$, between different simulations or data analysis scenarios and studied the distribution of these differences [boxplots with pairwise differences in prediction accuracy (by method) are provided in Fig. S3]. In analyses including markers, (either markers only or markers+QTL), adding QTL to the set of loci used to compute the **G** matrix increased prediction accuracy when Bayes A or Spike‐Slab were used, whereas the GBLUP methods did not benefit from having the QTL loci within the set of markers used to compute the **G** matrix. As expected, the prediction accuracy obtained in the RAND scenario was higher than the one obtained in the LOW‐MAF scenario; this pattern was observed across statistical methods.

**Figure 3:**
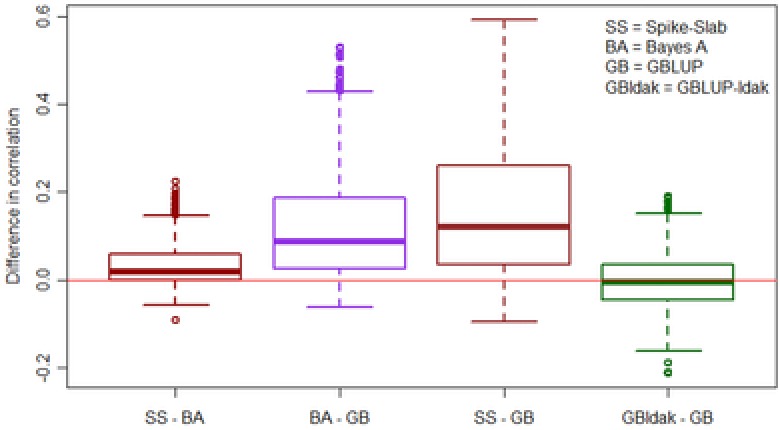
Pairwise difference in prediction accuracy (from simulation studies) across methods. Boxplots of the pairwise differences (across MC replicates and simulation scenarios) in prediction accuracy [correlation between predictions and simulated phenotypes $\mathrm{cor}(y,\hat{y})$] by pair of models.

Figure 3 gives boxplots of the differences in prediction accuracy by pair of models, across simulation scenarios. The Spike‐Slab models and Bayes A were significantly better than the GBLUP; the superiority of the Spike‐Slab over Bayes A was also systematic, but very small in magnitude.

## Results from Real Data Analysis

The estimates of genomic heritability and of prediction accuracy in testing data sets, averaged over 30 training‐testing partitions, are displayed in Table 3. The estimated genomic heritability ranged from 0.367 (Spike‐Slab) to 0.561 (GBLUP‐ldak). The GBLUP had an intermediate estimate of genomic heritability (0.435). Our estimates are in line with previous reports for human height using common SNPs (e.g., Yang et al., 2010; de los Campos et al., 2013b). These results are also in agreement with what we observed in the LOW‐MAF setting, in scenarios for traits without major QTL and using only marker genotypes for computing **G** (see Fig. 1D for *pve* = 0). The correlations between phenotypes and predictions were low (0.16–0.17) for all methods, and only slightly higher for the GBLUP methods. These correlations are in agreement with what we obtained in the simulation study in the LOW‐MAF scenario when QTL were not used in the model (see Fig. 2D).

**Table 3:** Estimates (SEs) of genomic heritability and of prediction accuracy (correlation between phenotypes and predictions in testing data sets) in real data analysis of human height

Method	Genomic heritability	Prediction accuracy^a^
Bayes A	0.494 (0.0001)	0.159 (0.044)
Spike‐Slab	0.367 (0.0005)	0.165 (0.043)
GBLUP	0.435 (0.0006)	0.169 (0.043)
GBLUP‐ldak	0.561 (0.004)	0.171 (0.041)

a Average correlation between predictions and phenotypes in testing data sets.

**Figure 4:**
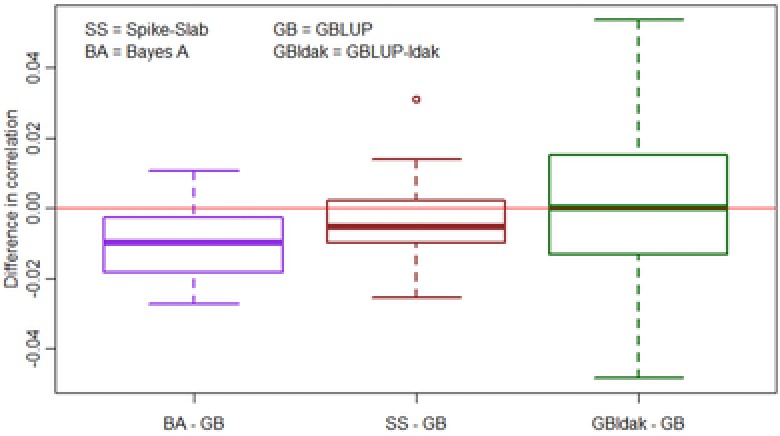
Difference in prediction accuracy (from real data analysis) across methods. Boxplots of the difference in prediction accuracy [correlation between predictions and phenotypes $\mathrm{cor}(y,\hat{y})$], within TRN–TST partition, between methods.

Figure 4 provides boxplots of the difference in prediction accuracy obtained, within each TRN–TST partition, between methods. Although the average difference in prediction accuracy between methods was small, the analysis of pair‐wise differences in prediction accuracy (by using the Wilcoxon signed rank test) suggested a statistically significant, albeit small, superiority of the GBLUP methods over Bayes A; the differences between the Spike‐Slab and GBLUP are nonsignificant.

### Discussion

In recent years, GWAS have found an unprecedented number of variants associated with important human traits and diseases (http://gds.nih.gov/). However, for complex traits and diseases, the variants identified so far usually explain a small fraction of inter‐individual differences in a trait or in disease risk, a problem referred to as the missing heritability of complex traits (Maher, 2008; Manolio et al., 2009; Eichler et al., 2010; Gibson, 2010; Makowsky et al., 2011). This problem has been partially attributed to the lack of power of GWAS to detect small‐effect variants, and some studies (e.g., Allen et al., 2010; Ober et al., 2012) have shown that the proportion of marker‐driven variance and prediction accuracy could be improved when prediction models include variants that show strong, but not GWAS‐significant association.

Several authors (e.g., de los Campos et al., 2010; Yang et al., 2010) have suggested the use of WGR methods (Meuwissen et al., 2001), where phenotypes are regressed on potentially hundreds of thousands of variants concurrently, for analysis and prediction of complex human traits and diseases. In human genetic applications, the most commonly used WGR method has been the GBLUP (Gondro et al., 2013). This method has been used primarily for the estimation of missing heritability (e.g., Eichler et al., 2010; Yang et al., 2010; Speed et al., 2012). Only a few studies have assessed these methods from a prediction perspective. These studies have reported poor prediction performance of GBLUP when training and validation samples were distantly related (e.g., de los Campos et al., 2013b). This leaves open the question of what avenues should be pursued to improve the prediction performance of WGR methods when used for the prediction of phenotypes for distantly related individuals.

The prediction accuracy of WGR is known to be affected by many important factors, including genetic relationship (e.g., VanRaden et al., 2009; Crossa et al., 2010), trait heritability (e.g., Hayes et al., 2009; Daetwyler et al., 2010), marker density (e.g., Vazquez et al., 2010; Makowsky et al., 2011; Ober et al., 2012; Erbe et al., 2013), the genetic architecture of the model (e.g., the number of QTL, the distribution of effects (VanRaden et al., 2009; Wimmer et al., 2013), the extent of LD between markers and QTL (Habier et al., 2007; Calus et al., 2008), the sample size (Hayes et al., 2009; Makowsky et al., 2011) and the method used (e.g., Habier et al., 2007; Hayes et al., 2009; VanRaden et al., 2009; Verbyla et al., 2009; Gao et al., 2013; Wimmer et al., 2013; Zhang et al., 2014). The vast majority of studies that have compared the predictive performance of shrinkage and variable selection methods have used family data from populations with intensive history of recent selection. Indeed, there has been little, if any, assessment of the factors that affect the prediction accuracy of WGRs using human data from distantly related individuals. In this article we contributed towards filling this gap by conducting an extensive simulation study where we assessed the impact on estimated missing heritability and on prediction accuracy of: (a) the extent of LD between markers and QTL, (b) the complexity of the trait architecture and (c) the statistical model used.

#### Missing heritability

Missing heritability can be attributed to imperfect LD between marker and QTL genotypes (e.g., Goddard & Hayes, 2009; Yang et al., 2010; de los Campos et al., 2013b). Therefore, in scenarios where QTL genotypes were used for analysis (either when QTL only or when both markers and QTL were used) there is no missing heritability because the causal loci were included in the set of genotypes used for data analysis. In these analysis scenarios (only QTL or markers and QTL), estimates of genomic heritability above or below the simulated heritability (0.5) reflect bias of the estimation method.

When the analysis was carried out using QTL genotypes only, the Spike‐Slab and GBLUP methods yielded estimates very close to the simulated heritability, while Bayes A and GBLUP‐ldak yielded substantial biases. In the case of Bayes A, the estimate was downwardly biased in scenarios where a few QTL made a substantial contribution to genetic variance (e.g., *p* = 50, *pve* = 0.75) and GBLUP‐ldak showed a clearly downwardly biased estimate in the RAND scenario.

When markers and QTL were used for analysis, the results differed between the RAND and LOW‐MAF scenarios. In the RAND scenario, GBLUP and Spike‐Slab yielded almost unbiased estimates, while Bayes A and GBLUP‐ldak yielded upwardly biased estimates under simple genetic architectures. In the LOW‐MAF scenario, GBLUP, Spike‐Slab and Bayes A yielded downwardly biased estimates whereas estimates from GBLUP‐ldak were slightly biased upward.

Finally, in scenarios using only markers the estimated genomic heritability was very close to the trait heritability in the RAND scenario, whereas in the LOW‐MAF scenario estimates revealed a substantial extent of missing heritability.

The observation that having a different distribution of allele frequencies at markers and at QTL can induce a large extent of missing heritability is in line with the reasoning and results presented in some studies (Goldstein, 2009; Yang et al., 2010; Lee et al., 2012; de los Campos et al., 2013b). This result is also in agreement with the fact that the extent of LD between markers and QTL in the LOW‐MAF scenarios was much weaker than in the RAND scenarios (see Table 2). It should be noted that in all simulation scenarios considered in our study, including the LOW‐MAF scenario, the frequency of rare variants among the QTL was limited relative to what one could have with sequence data, because the genotypes used in our study were all obtained from a panel of common SNPs. Therefore, one could speculate that the extent of differences in distribution of allele frequency between markers and causal loci and the corresponding extent of missing heritability may be even more extreme with real phenotypes than the one observed in our LOW‐MAF scenario.

Importantly, within any scenario we found remarkable differences in estimates of genomic heritability across models, and there was no single method with smallest bias across all genetic architectures and analysis scenarios (QTL, markers+QTL, or only markers). The GBLUP and Spike‐Slab methods performed well in the RAND scenario, but had clear problems in the LOW‐MAF scenarios (both had seriously downwardly biased estimates in the analysis based on markers and QTL). However, GBLUP‐ldak exhibited some clear problems in the RAND scenarios (downwardly biased estimates when analysis was based on QTL only) or upwardly biased estimates in the LOW‐MAF analysis based on markers and QTL). Finally, Bayes A showed somewhat erratic behaviour, especially with simple genetic architectures (e.g., *p* = 50, *pve* = 0.75); we believe that this is not a limitation of the model *per se* but a consequence of the degree‐of‐freedom parameter being fixed. Estimating this parameter from the data, as done, for instance in Yi and Xu (2008), is likely to confer more flexibility to Bayes A to cope with different genetic architectures.

### Prediction accuracy

When the analysis was carried out using only QTL genotypes (“perfect LD,” Fig. 2C and F), all methods achieved relatively high prediction accuracy (correlation of about 0.5 or greater, i.e., an *R* ^2^ 50% or more of the trait heritability). This indicates that if one is able to narrow down the influential genetic regions of a trait to a limited number (5000 loci in our simulation), regularised regressions like the one used here can yield relatively high prediction accuracy. In these scenarios, the prediction accuracy of the GBLUP and GBLUP‐ldak methods was not affected by the genetic architecture and tended to be poorer than that of Bayes A and the Spike‐Slab methods. Bayes A and Spike‐Slab performed similarly and clearly better than any of the GBLUP methods in scenarios where a limited number of QTL (e.g., 50 or 250) explained a sizable proportion of the genetic variance. However, with increase in trait complexity there was a decrease in prediction performance of these two methods, to the point that the three methods performed very similarly when the most complex genetic architecture was considered (5,000 QTL without any “major effect” one). Overall, our results are in agreement with previous studies in animal and plant breeding (Daetwyler et al., 2010; Wimmer et al., 2013) that have reported that: (a) the prediction accuracy of GBLUP is largely independent of the genetic architecture of the trait, and (b) with simple genetic architectures there are benefits of using methods such as Bayes B, Spike‐Slab, Bayes C, or Bayes A, relative to ridge‐regression type‐methods. However, as the trait architecture became more complex, these differences disappeared.

#### When markers and QTL were jointly used

When markers and QTL were jointly used (Fig. 2B and E) or when only markers were used (Fig. 2A and D), important changes in prediction accuracy were observed. The prediction accuracy of any of the GBLUP methods was reduced from correlation levels of the order of 0.45 (QTL‐only analysis) to 0.15 when both markers and QTL were used, and to levels below 0.1 when only markers were used. This reflects the limitations of using methods such as GBLUP or GBLUP‐ldak where the effects of all predictors are homogeneously shrunk, especially in situations where a large number of markers do not have effects.

In scenarios where 50 or 250 QTL explained a sizable proportion (e.g., 0.75) of the genetic variance, the benefits of using methods that perform variable selection (Spike‐Slab) or differential shrinkage of estimated effects (Bayes A) relative to the GBLUP methods were pronounced. In the scenario with the simplest genetic architecture (50 QTL explaining 75% of the genetic variance) these methods, especially the Spike‐Slab were able to achieve levels of prediction accuracy comparable to those obtained when only QTL genotypes were used, illustrating the “oracle” property (e.g., Ishwaran & Rao, 2005; Scheipl et al., 2013) that these methods have. However, as the complexity of the trait increased, the predictive performance of these methods decreased and in the most complex scenario (5000 small QTL) all methods performed similarly.

#### Real data analysis

Human height is believed to be a trait affected by a very large number of small‐effect QTL (e.g., Allen et al., 2010; Yang et al., 2010). The analysis conducted with human height data from the GENEVA data set very closely matched the results from the simulation for scenarios with large numbers of small effect QTL, where the distributions of allele frequency at markers and at QTL were different. We estimated a sizable proportion of missing heritability, given a trait heritability of 0.8, the estimates of missing heritability ranged from 0.24 with GBLUP‐ldak to 0.54 with Spike‐Slab and very poor prediction accuracy (correlation of about 0.16–0.17, and very similar across methods).

### Implications

The results presented in this study have several implications. First, estimates of missing heritability derived from distantly related individuals using WGR methods need to be treated with caution. Although they are indicative of how imperfect LD between markers and QTL can limit the ability of a model to capture the genetic signal, some of the results presented here indicate that under some circumstances estimates can have a sizable bias. In addition, we observed that in some scenarios these estimates of heritability can vary significantly between methods. This is not surprising because the proportion of variance explained by a model depends both on the input information (markers/QTL, etc.) and on the statistical model used. We believe that this model‐genetic architecture dependency has been overlooked so far. Importantly, the model that yields the highest estimated genomic heritability is not necessarily the one that yields the best prediction accuracy.

Second, the assessment of prediction accuracy suggests that for traits in which a limited number of regions explain a sizable proportion of genetic variance, the use of WGR methods that perform variable selection or differential shrinkage of estimates of effects is strongly recommended over ridge‐regression type methods such as the GBLUP. However, for very complex traits such as human height, all the methods evaluated yield low prediction accuracy. It remains to be determined whether significant increases in sample size (which likely should be by orders of magnitude) will also yield substantial gains in prediction accuracy.

## Supporting information

Disclaimer: Supplementary materials have been peer‐reviewed but not copyedited.

**Figure S1** Prior distributions commonly used in Bayesian regression models.

**Figure S2** *R* ^2^ statistic in training data sets.

**Figure S3** Difference in prediction accuracy by scenario and data used.

**Figure S4** Differences between GBLUP methods in the analysis of human height.

**Table S1** Genomic heritability estimates obtained with the GBLUP method in the RAND scenario by: genetic architecture simulated, data used and Monte Carlo replicate.

**Table S2** Genomic heritability estimates obtained with the GBLUP method in the LOW‐MAF scenario by: genetic architecture simulated. data used and Monte Carlo replicate.

**Table S3** Average (SD, both across Monte Carlo replicates) correlation between simulated phenotype and predictions in Training data sets by method, simulation scenario and data used for the analysis.

**Table S4** Average (SD, both across 30 replicates) *R* ^2^ in validation data sets by simulation scenario, data used for analysis and estimation method.

**Table S5** Correlation and *R* ^2^ between human height and genomic predictions in testing data sets by method and testing set.
