# Supplementary figures and images for: Effectiveness of Shrinkage and Variable Selection Methods for the Prediction of Complex Human Traits using Data from Distantly Related Individuals
Source: Ann Hum Genet. 2015 Jan 20;79(2):122–35. doi: 10.1111/ahg.12099 (PMC4428155; doi:10.1111/ahg.12099)

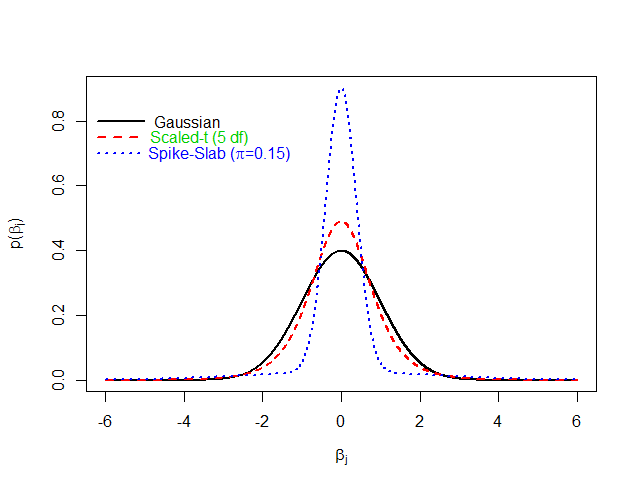

Supplement: Supplementary file 1 — Figure S1 Prior distributions commonly used in Bayesian regression models. [file AHG-79-122-s001.tiff]

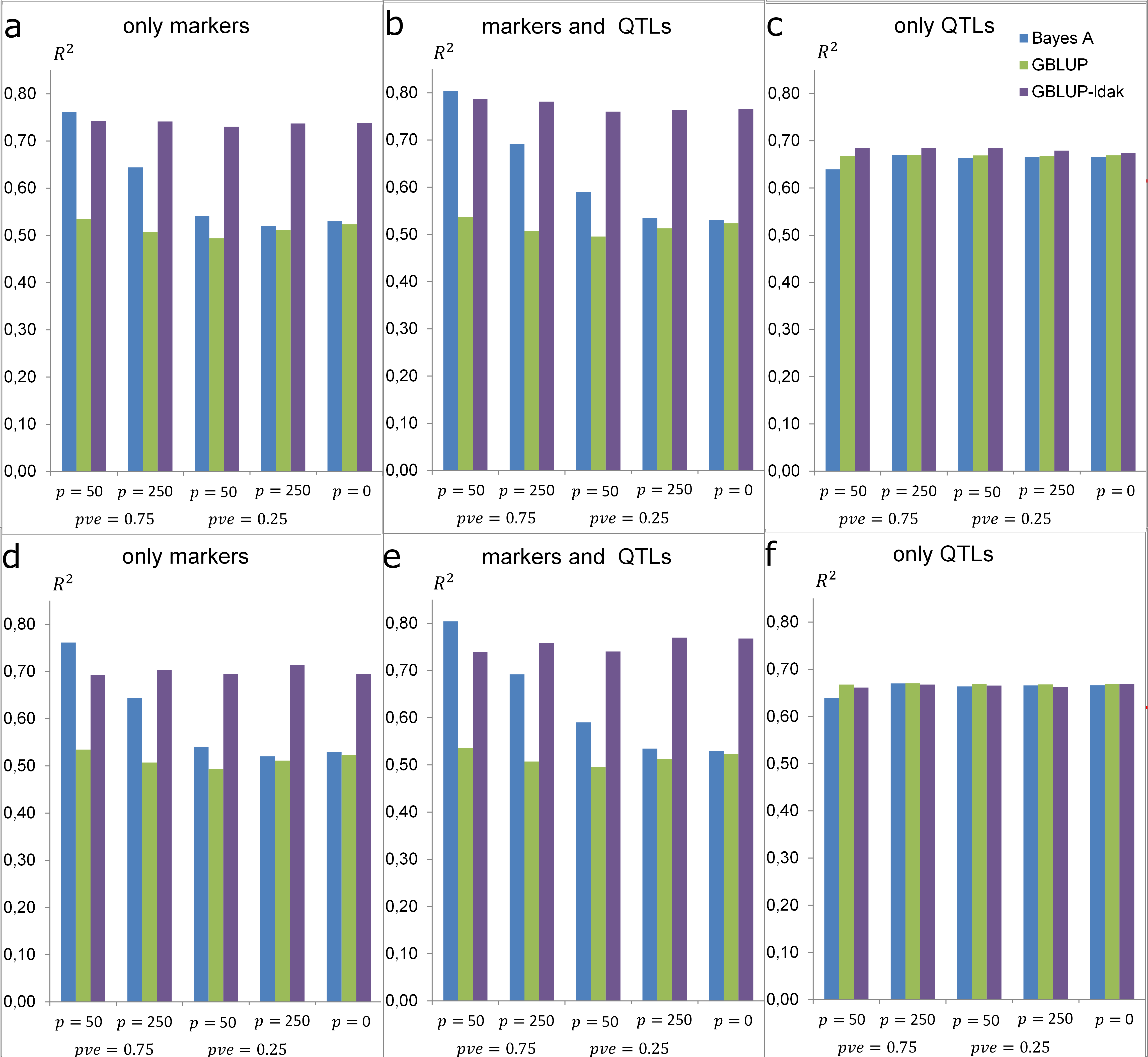

Supplement: Supplementary file 2 — Figure S2 R 2 statistic in training data sets. [file AHG-79-122-s002.tif]

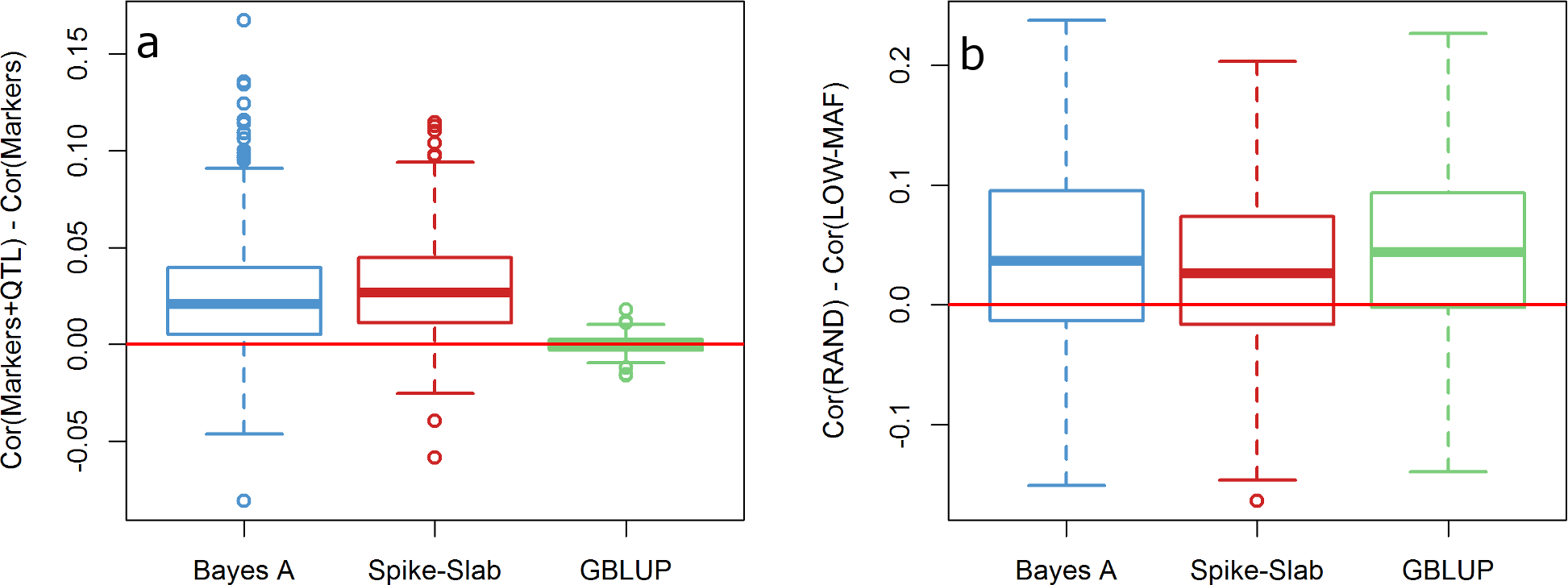

Supplement: Supplementary file 3 — Figure S3 Difference in prediction accuracy by scenario and data used. [file AHG-79-122-s003.tif]
