# Supplementary material for: Effectiveness of Shrinkage and Variable Selection Methods for the Prediction of Complex Human Traits using Data from Distantly Related Individuals
Source: Ann Hum Genet. 2015 Jan 20;79(2):122–35. doi: 10.1111/ahg.12099 (PMC4428155; doi:10.1111/ahg.12099)
Supplement: Supplementary file 5 — Table S1 Genomic heritability estimates obtained with the GBLUP method in the RAND scenario by: genetic architecture simulated, data used and Monte Carlo replicate. [file AHG-79-122-s005.doc]

**Table S1.** Genomic heritability estimates obtained with the GBLUP method in the RAND scenario by: genetic architecture simulated. data used. and Monte Carlo replicate.

| Data used | only markers | | | | | markers and QTLs | | | | | only QTLs | | | | |
| --- | --- | --- | --- | --- | --- | --- | --- | --- | --- | --- | --- | --- | --- | --- | --- |
| LEQTLs* | 50 | | 250 | | none | 50 | | 250 | | none | 50 | | 250 | | none |
| pve** | 25% | 75% | 25% | 75% | - | 25% | 75% | 25% | 75% | - | 25% | 75% | 25% | 75% | - |
| run 1 | 0.586556514 | 0.430165065 | 0.61534473 | 0.493293641 | 0.535774809 | 0.594010795 | 0.440808483 | 0.627343804 | 0.498904397 | 0.549734952 | 0.49061564 | 0.50412256 | 0.4957092 | 0.53454544 | 0.4953998 |
| run 2 | 0.532878365 | 0.535588053 | 0.552418637 | 0.585671207 | 0.588604479 | 0.535157921 | 0.543927303 | 0.555975249 | 0.58765338 | 0.592787168 | 0.50907393 | 0.47890885 | 0.47667901 | 0.51216488 | 0.48740111 |
| run 3 | 0.429712912 | 0.644683435 | 0.589326155 | 0.411636484 | 0.37882413 | 0.438185728 | 0.656054181 | 0.59111111 | 0.41296188 | 0.382026751 | 0.52968702 | 0.50144414 | 0.51530577 | 0.51293921 | 0.46941136 |
| run 4 | 0.551654119 | 0.420781005 | 0.505117917 | 0.542886393 | 0.624399149 | 0.548180186 | 0.427841378 | 0.510394903 | 0.550498468 | 0.629963733 | 0.50174254 | 0.50439296 | 0.49871179 | 0.48256575 | 0.5233572 |
| run 5 | 0.532071381 | 0.503645043 | 0.478013045 | 0.475579506 | 0.522816677 | 0.546051544 | 0.515827688 | 0.490463897 | 0.485759513 | 0.526819968 | 0.47921724 | 0.50579434 | 0.47390503 | 0.49338593 | 0.48323192 |
| run 6 | 0.510328798 | 0.643230314 | 0.536178427 | 0.498495824 | 0.510575185 | 0.510818157 | 0.640165684 | 0.533808932 | 0.497748864 | 0.517456677 | 0.50827645 | 0.4990104 | 0.51706735 | 0.49148487 | 0.49607594 |
| run 7 | 0.500387731 | 0.523722982 | 0.529125937 | 0.514875318 | 0.489297783 | 0.500365728 | 0.527057049 | 0.5438832 | 0.51852223 | 0.493352574 | 0.51055052 | 0.50287882 | 0.50213576 | 0.49039096 | 0.50110716 |
| run 8 | 0.552981481 | 0.397704464 | 0.564334436 | 0.529853334 | 0.507716108 | 0.556335863 | 0.404389037 | 0.570610802 | 0.541423028 | 0.512383334 | 0.49173748 | 0.47443241 | 0.49582191 | 0.50513994 | 0.51214961 |
| run 9  run 10 | 0.509165237 | 0.444221927 | 0.444114286 | 0.491722644 | 0.546386383 | 0.520815956 | 0.453529041 | 0.4527447 | 0.49379298 | 0.5528077 | 0.53733266 | 0.49725346 | 0.50921313 | 0.49812408 | 0.49034242 |
| run 10 | 0.473066408 | 0.645908944 | 0.432266926 | 0.637440863 | 0.565983368 | 0.490477327 | 0.65829032 | 0.431811964 | 0.643445348 | 0.577799021 | 0.52963943 | 0.52898773 | 0.46457109 | 0.51933323 | 0.52117257 |
| run 11 | 0.515784681 | 0.536260168 | 0.43565657 | 0.47998536 | 0.497602244 | 0.520591635 | 0.530138994 | 0.440569411 | 0.487704188 | 0.505435145 | 0.49649229 | 0.49441019 | 0.45215274 | 0.49566828 | 0.45196404 |
| run 12 | 0.492357307 | 0.539361235 | 0.377136996 | 0.607053627 | 0.554533846 | 0.502244082 | 0.54548506 | 0.384796671 | 0.616877306 | 0.566646174 | 0.47086543 | 0.50557337 | 0.51985507 | 0.50879779 | 0.52039312 |
| run 13 | 0.414906208 | 0.659945713 | 0.635000249 | 0.549013002 | 0.510385883 | 0.41992123 | 0.666594674 | 0.641820078 | 0.55153739 | 0.514292495 | 0.51851759 | 0.49954223 | 0.51738194 | 0.49486985 | 0.50761809 |
| run 14 | 0.502956634 | 0.441864078 | 0.639691538 | 0.417998935 | 0.517662939 | 0.507655713 | 0.44146757 | 0.646895675 | 0.414456753 | 0.523035371 | 0.50857392 | 0.53548298 | 0.50694205 | 0.50335022 | 0.49023897 |
| run 15 | 0.499071181 | 0.469830123 | 0.481234711 | 0.533747056 | 0.617445638 | 0.497145879 | 0.480855758 | 0.492352653 | 0.534400016 | 0.629389865 | 0.49691849 | 0.52102035 | 0.5258806 | 0.50932609 | 0.50780268 |
| run 16 | 0.462125164 | 0.514673258 | 0.535006875 | 0.482850407 | 0.517368481 | 0.467103434 | 0.522365107 | 0.5403794 | 0.491953307 | 0.52769445 | 0.50588698 | 0.51391545 | 0.4940292 | 0.4976533 | 0.49217763 |
| run 17 | 0.522805805 | 0.429712885 | 0.499656611 | 0.394532841 | 0.516839084 | 0.534848208 | 0.435419031 | 0.50274494 | 0.406355324 | 0.518580857 | 0.50440984 | 0.48522168 | 0.47219512 | 0.50362118 | 0.48705808 |
| run 18 | 0.514248359 | 0.603082298 | 0.441562185 | 0.512763335 | 0.683392372 | 0.519580131 | 0.614275843 | 0.445281589 | 0.527684713 | 0.695110618 | 0.48777495 | 0.50746709 | 0.50102491 | 0.49692261 | 0.5212846 |
| run 19 | 0.515460366 | 0.569546017 | 0.4643852 | 0.530689429 | 0.575616133 | 0.522750496 | 0.59457233 | 0.474951499 | 0.535267929 | 0.582959152 | 0.51135694 | 0.49929126 | 0.49396446 | 0.49417331 | 0.50465994 |
| run 20 | 0.618622778 | 0.503493464 | 0.502993127 | 0.490925155 | 0.541799809 | 0.63800879 | 0.499346064 | 0.515184015 | 0.487313215 | 0.546370099 | 0.47877609 | 0.51309887 | 0.51451389 | 0.5217886 | 0.48813755 |
| run 21 | 0.422644712 | 0.579822973 | 0.610268026 | 0.489033929 | 0.54590442 | 0.434566655 | 0.578924182 | 0.622482529 | 0.489514231 | 0.557479526 | 0.473047 | 0.49727442 | 0.48821514 | 0.48944367 | 0.51082585 |
| run 22 | 0.512253427 | 0.501145787 | 0.452627005 | 0.457498325 | 0.624852415 | 0.522662469 | 0.513197195 | 0.451948791 | 0.46196072 | 0.635366171 | 0.49880501 | 0.50898445 | 0.50340309 | 0.48651929 | 0.51221754 |
| run 23 | 0.45262392 | 0.396117017 | 0.472878013 | 0.545553375 | 0.532645066 | 0.453488964 | 0.38903127 | 0.485366898 | 0.555551714 | 0.530858969 | 0.49402892 | 0.48937659 | 0.51480298 | 0.50405976 | 0.53420784 |
| run 24 | 0.371767456 | 0.621747302 | 0.490574783 | 0.57861373 | 0.424274367 | 0.374236878 | 0.630037352 | 0.49164256 | 0.57722328 | 0.427637299 | 0.49047417 | 0.47022492 | 0.51766876 | 0.49636542 | 0.51836157 |
| run 25 | 0.504572212 | 0.546323115 | 0.453924351 | 0.572814641 | 0.488241956 | 0.50317068 | 0.545124779 | 0.465122283 | 0.577480295 | 0.500521339 | 0.47700581 | 0.52082734 | 0.48453761 | 0.48524768 | 0.51545363 |
| run 26 | 0.491624285 | 0.431296286 | 0.676321871 | 0.444593764 | 0.461792778 | 0.496496259 | 0.431784424 | 0.686277912 | 0.438831414 | 0.468327392 | 0.50292612 | 0.50800267 | 0.50188153 | 0.51105642 | 0.49876828 |
| run 27 | 0.536054646 | 0.503782894 | 0.490444521 | 0.625102824 | 0.409572743 | 0.554110177 | 0.503671438 | 0.494497055 | 0.62497654 | 0.419909728 | 0.4949107 | 0.50856891 | 0.52032801 | 0.50963975 | 0.51086595 |
| run 28 | 0.536661038 | 0.478214901 | 0.626184409 | 0.424578829 | 0.436008464 | 0.546111187 | 0.48795188 | 0.632395184 | 0.441961817 | 0.435731462 | 0.50944094 | 0.51262312 | 0.51282067 | 0.49451086 | 0.48633103 |
| run 29 | 0.529944466 | 0.478392435 | 0.446393452 | 0.391374095 | 0.426683258 | 0.534294946 | 0.481780575 | 0.447722483 | 0.399932391 | 0.426048612 | 0.53466684 | 0.48403406 | 0.51332571 | 0.5093672 | 0.51258207 |
| run 30 | 0.5303485 | 0.609714022 | 0.465035405 | 0.488571146 | 0.486494905 | 0.531675347 | 0.610283021 | 0.477074753 | 0.508370312 | 0.49304516 | 0.50704478 | 0.51915201 | 0.50318181 | 0.4848312 | 0.51124067 |
| average | 0.504 | 0.520 | 0.515 | 0.507 | 0.521 | 0.511 | 0.526 | 0.522 | 0.512 | 0.528 | 0.50165986 | 0.50304392 | 0.50024084 | 0.50124289 | 0.50206127 |
| sd | 0.050 | 0.078 | 0.074 | 0.064 | 0.067 | 0.051 | 0.079 | 0.075 | 0.064 | 0.069 | 0.017 | 0.015 | 0.018 | 0.012 | 0.017 |

*: Number of Large Effect QTL **: % of Genetic Variance Explained by Large Effect QTL
