# Supplementary material for: Effectiveness of Shrinkage and Variable Selection Methods for the Prediction of Complex Human Traits using Data from Distantly Related Individuals
Source: Ann Hum Genet. 2015 Jan 20;79(2):122–35. doi: 10.1111/ahg.12099 (PMC4428155; doi:10.1111/ahg.12099)
Supplement: Supplementary file 6 — Table S2 Genomic heritability estimates obtained with the GBLUP method in the LOW‐MAF scenario by: genetic architecture simulated. data used and Monte Carlo replicate. [file AHG-79-122-s006.doc]

**Table S2.** Genomic heritability estimates obtained with the GBLUP method in the LOW-MAF scenario by: genetic architecture simulated. data used. and Monte Carlo replicate.

| Data used | only markers | | | | | markers and QTLs | | | | | only QTLs | | | | |
| --- | --- | --- | --- | --- | --- | --- | --- | --- | --- | --- | --- | --- | --- | --- | --- |
| LEQTLs* | 50 | | 250 | | none | 50 | | 250 | | none | 50 | | 250 | | none |
| pve** | 25% | 75% | 25% | 75% | - | 25% | 75% | 25% | 75% | - | 25% | 75% | 25% | 75% | - |
| run 1 | 0.34699282 | 0.19930061 | 0.32733194 | 0.32719934 | 0.28150596 | 0.34270392 | 0.19725232 | 0.32846111 | 0.33333278 | 0.28722224 | 0.51116114 | 0.48537207 | 0.45786239 | 0.46237448 | 0.4705372 |
| run 2 | 0.35631155 | 0.28458025 | 0.25359844 | 0.25884187 | 0.36694559 | 0.35194403 | 0.27910011 | 0.25332687 | 0.2530924 | 0.37237036 | 0.45517372 | 0.47784756 | 0.4783433 | 0.49981991 | 0.46986616 |
| run 3 | 0.20020696 | 0.38122198 | 0.32772968 | 0.20038752 | 0.3359239 | 0.19901987 | 0.3837398 | 0.33663835 | 0.20223206 | 0.33363561 | 0.49264315 | 0.47398075 | 0.50877669 | 0.48982018 | 0.480463 |
| run 4 | 0.32155404 | 0.32740804 | 0.29430457 | 0.30349817 | 0.41620535 | 0.32502859 | 0.33418458 | 0.29296061 | 0.30812867 | 0.41316008 | 0.5041648 | 0.47064877 | 0.49802485 | 0.49806876 | 0.48421188 |
| run 5 | 0.27071174 | 0.29990706 | 0.27254354 | 0.3944054 | 0.29308545 | 0.27202982 | 0.30286739 | 0.27291504 | 0.39623297 | 0.29176924 | 0.47586412 | 0.5055277 | 0.46459275 | 0.49738236 | 0.4984041 |
| run 6 | 0.36399508 | 0.40016332 | 0.34787004 | 0.32027554 | 0.23659994 | 0.36796728 | 0.4071976 | 0.34598479 | 0.32549253 | 0.24048532 | 0.51488388 | 0.47818821 | 0.48410997 | 0.48673588 | 0.52064382 |
| run 7 | 0.27473164 | 0.32965447 | 0.33662996 | 0.37206514 | 0.37598444 | 0.27045368 | 0.33167003 | 0.33527546 | 0.37356089 | 0.37362156 | 0.51858493 | 0.48317379 | 0.52191596 | 0.50151715 | 0.50591691 |
| run 8 | 0.3679466 | 0.23017341 | 0.26408454 | 0.35086145 | 0.46239264 | 0.36813687 | 0.23087061 | 0.26500749 | 0.34995429 | 0.46311269 | 0.49969373 | 0.45575656 | 0.48310283 | 0.49758258 | 0.49503804 |
| run 9  run 10 | 0.2574716 | 0.30860286 | 0.38913442 | 0.27017756 | 0.27421757 | 0.25977949 | 0.31358466 | 0.39471441 | 0.27026424 | 0.26988441 | 0.52454733 | 0.51598698 | 0.48599112 | 0.47840411 | 0.49577288 |
| run 10 | 0.31308347 | 0.4413169 | 0.29858834 | 0.26412079 | 0.3255947 | 0.31330936 | 0.44735789 | 0.29562628 | 0.26333518 | 0.32073231 | 0.49568066 | 0.49819319 | 0.47279836 | 0.48917119 | 0.4700515 |
| run 11 | 0.2767726 | 0.27358459 | 0.27612744 | 0.21309822 | 0.24853633 | 0.27529882 | 0.27123733 | 0.28273967 | 0.20779087 | 0.24757782 | 0.48863516 | 0.4841903 | 0.4791013 | 0.49061715 | 0.48204722 |
| run 12 | 0.26638403 | 0.33886647 | 0.27536071 | 0.2899249 | 0.43542769 | 0.26683797 | 0.33914233 | 0.2762871 | 0.29315209 | 0.43601541 | 0.46059732 | 0.49558713 | 0.47120516 | 0.48198577 | 0.49264602 |
| run 13 | 0.28870077 | 0.26944965 | 0.45107502 | 0.32200115 | 0.25546126 | 0.29147075 | 0.27646083 | 0.45072612 | 0.31999633 | 0.25043858 | 0.49216917 | 0.48270237 | 0.46982459 | 0.43961471 | 0.49138942 |
| run 14 | 0.28660369 | 0.34842273 | 0.24931526 | 0.29307828 | 0.42876119 | 0.28697152 | 0.35042875 | 0.25150499 | 0.29056211 | 0.42785016 | 0.47826905 | 0.47890493 | 0.48239746 | 0.46753643 | 0.5116582 |
| run 15 | 0.26231625 | 0.34122918 | 0.33925117 | 0.34870059 | 0.42466427 | 0.25830359 | 0.34086254 | 0.3314589 | 0.34256495 | 0.41689712 | 0.4729956 | 0.47034556 | 0.49971522 | 0.50447431 | 0.50282972 |
| run 16 | 0.22406491 | 0.29368005 | 0.37966083 | 0.26926087 | 0.33203687 | 0.22439924 | 0.30185028 | 0.3705569 | 0.26803665 | 0.34363646 | 0.51073138 | 0.51258413 | 0.48333117 | 0.469954 | 0.47593695 |
| run 17 | 0.29828284 | 0.35133379 | 0.24441137 | 0.41002083 | 0.23007285 | 0.29764121 | 0.35820987 | 0.2456361 | 0.40799901 | 0.22973039 | 0.47719608 | 0.484735 | 0.48719542 | 0.48823008 | 0.45463644 |
| run 18 | 0.25891033 | 0.27472332 | 0.22610083 | 0.32798182 | 0.28816286 | 0.26853358 | 0.27512093 | 0.23521582 | 0.33080698 | 0.28490383 | 0.48279604 | 0.50271717 | 0.50626038 | 0.48942554 | 0.44372421 |
| run 19 | 0.282317 | 0.34462347 | 0.23920054 | 0.27567273 | 0.27728298 | 0.28528489 | 0.34256341 | 0.24285787 | 0.27745596 | 0.27949331 | 0.49671299 | 0.46710462 | 0.46579321 | 0.4613128 | 0.49005432 |
| run 20 | 0.38722866 | 0.52163032 | 0.2683161 | 0.22810203 | 0.2928362 | 0.38903372 | 0.51852171 | 0.27342891 | 0.23572922 | 0.29154856 | 0.4881436 | 0.50317919 | 0.47708718 | 0.49620819 | 0.47280399 |
| run 21 | 0.34984304 | 0.36892556 | 0.41364946 | 0.35765179 | 0.28257243 | 0.35087444 | 0.36806976 | 0.41086377 | 0.35008161 | 0.28913785 | 0.51835335 | 0.48043452 | 0.50362935 | 0.4506835 | 0.47102915 |
| run 22 | 0.22788359 | 0.32302052 | 0.23910139 | 0.37239243 | 0.40621801 | 0.22456563 | 0.32073446 | 0.24527644 | 0.37879727 | 0.40892954 | 0.46588771 | 0.48152634 | 0.48006057 | 0.48202044 | 0.5057055 |
| run 23 | 0.22926949 | 0.25636147 | 0.36279317 | 0.33258598 | 0.42499204 | 0.23146207 | 0.26169733 | 0.3661877 | 0.32716081 | 0.42270528 | 0.47061446 | 0.45723318 | 0.45411401 | 0.49875747 | 0.49333469 |
| run 24 | 0.23851454 | 0.31649712 | 0.27451648 | 0.30870052 | 0.19604434 | 0.2454586 | 0.31274961 | 0.27260694 | 0.30633136 | 0.19649718 | 0.47182789 | 0.4766263 | 0.5031382 | 0.47648551 | 0.46387244 |
| run 25 | 0.28636921 | 0.30790327 | 0.27400975 | 0.29441468 | 0.34285294 | 0.28868246 | 0.30951866 | 0.27366147 | 0.29567795 | 0.3398191 | 0.49466541 | 0.47945032 | 0.48570879 | 0.47951021 | 0.48973852 |
| run 26 | 0.36470437 | 0.4012252 | 0.40127401 | 0.27482789 | 0.33792855 | 0.36428286 | 0.39751516 | 0.40001721 | 0.27609077 | 0.33578571 | 0.4741047 | 0.47168797 | 0.46933229 | 0.50628697 | 0.48469993 |
| run 27 | 0.3897106 | 0.26882868 | 0.36506078 | 0.34007722 | 0.24992236 | 0.39392719 | 0.26854553 | 0.359752 | 0.34254315 | 0.25242023 | 0.48061822 | 0.48431628 | 0.48566341 | 0.48622502 | 0.48479049 |
| run 28 | 0.33505686 | 0.34060309 | 0.34670365 | 0.31500015 | 0.26021422 | 0.34178245 | 0.33849278 | 0.35033472 | 0.31694703 | 0.26018945 | 0.48161988 | 0.47902291 | 0.49744854 | 0.5128495 | 0.47243208 |
| run 29 | 0.27523014 | 0.34207859 | 0.35011792 | 0.31934604 | 0.3044072 | 0.27384967 | 0.34227394 | 0.34602872 | 0.31678799 | 0.30003737 | 0.47215508 | 0.46074178 | 0.47862675 | 0.5037664 | 0.44900258 |
| run 30 | 0.31871714 | 0.35636937 | 0.22909385 | 0.24445663 | 0.23139135 | 0.31906429 | 0.35671849 | 0.23149308 | 0.24049152 | 0.23361919 | 0.47431514 | 0.50315083 | 0.4939736 | 0.47485852 | 0.51755195 |
| average | 0.29732952 | 0.32805618 | 0.31056517 | 0.30663758 | 0.32060805 | 0.29826993 | 0.32928462 | 0.3112515 | 0.30668766 | 0.32044088 | 0.48816019 | 0.48336388 | 0.48430416 | 0.48538931 | 0.48469298 |
| sd | 0.051 | 0.062 | 0.060 | 0.050 | 0.072 | 0.051 | 0.062 | 0.058 | 0.051 | 0.072 | 0.018 | 0.015 | 0.015 | 0.017 | 0.019 |

*: Number of Large Effect QTL **: % of Genetic Variance Explained by Large Effect QTL
