# Supplementary material for: Effectiveness of Shrinkage and Variable Selection Methods for the Prediction of Complex Human Traits using Data from Distantly Related Individuals
Source: Ann Hum Genet. 2015 Jan 20;79(2):122–35. doi: 10.1111/ahg.12099 (PMC4428155; doi:10.1111/ahg.12099)
Supplement: Supplementary file 7 — Table S3 Average (SD, both across Monte Carlo replicates) correlation between simulated phenotype and predictions in Training data sets by method, simulation scenario and data used for the analysis. [file AHG-79-122-s007.doc]

**Table S3**. Average (SD, both across Monte Carlo replicates) correlation between simulated phenotype and predictions in Training data sets by method, simulation scenario and data used for the analysis.

| Simulation Scenarios | | | Data Analysis Method & Information Used . | | | | | | | | |
| --- | --- | --- | --- | --- | --- | --- | --- | --- | --- | --- | --- |
| Number of Large Effect QTL | % of Genetic Variance Explained by Large Effect QTL | Sampl-ing of QTL | GBLUP . | | | BayesA . | | | Spike-Slab . | | |
|  |  |  | Markers | Markers+QTL | QTL | Markers | Markers+QTL | QTL | Markers | Markers+QTL | QTL |
| 50 | 25 | UNIF | 0.174 (0.04) | 0.174  (0.04) | 0.463  (0.04) | 0.283  (0.06) | 0.303  (0.05) | 0.513  (0.05) | 0.309  (0.05) | 0.331  (0.05) | 0.513  (0.04) |
|  |  | LOW-MAF | 0.104  (0.05) | 0.104  (0.05) | 0.447  (0.04) | 0.200  (0.08) | 0.236  (0.05) | 0.516  (0.04) | 0.268  (0.05) | 0.305  (0.05) | 0.504  (0.04) |
|  | 75 | UNIF | 0.147  (0.05) | 0.147  (0.05) | 0.442  (0.05) | 0.512  (0.04) | 0.547  (0.04) | 0.604  (0.04) | 0.554  (0.04) | 0.581  (0.03) | 0.601  (0.03) |
|  |  | LOW-MAF | 0.085  (0.04) | 0.085  (0.04) | 0.427  (0.04) | 0.473  (0.05) | 0.524  (0.04) | 0.607  (0.03) | 0.528  (0.04) | 0.570  (0.03) | 0.601  (0.03) |
| 250 | 25 | UNIF | 0.158  (0.05) | 0.157  (0.05) | 0.459  (0.04) | 0.178  (0.06) | 0.193  (0.05) | 0.492  (0.04) | 0.209  (0.06) | 0.227  (0.05) | 0.488  (0.04) |
|  |  | LOW-MAF | 0.086  (0.05) | 0.085  (0.05) | 0.429  (0.04) | 0.110  (0.05) | 0.111  (0.06) | 0.485  (0.05) | 0.155  (0.06) | 0.175  (0.06) | 0.465  (0.04) |
|  | 75 | UNIF | 0.153  (0.04) | 0.154  (0.04) | 0.434  (0.04) | 0.330  (0.06) | 0.376  (0.05) | 0.550  (0.04) | 0.443  (0.05) | 0.483  (0.05) | 0.565  (0.04) |
|  |  | LOW-MAF | 0.105  (0.04) | 0.106  (0.04) | 0.440  (0.04) | 0.286  (0.05) | 0.325  (0.03) | 0.564  (0.04) | 0.420  (0.05) | 0.470  (0.04) | 0.564  (0.04) |
| None | --- | UNIF | 0.155  (0.06) | 0.153  (0.06) | 0.445  (0.05) | 0.143  (0.07) | 0.162  (0.06) | 0.447  (0.04) | 0.161  (0.06) | 0.179  (0.06) | 0.464  (0.05) |
|  |  | LOW-MAF | 0.094  (0.05) | 0.094  (0.05) | 0.449  (0.04) | 0.069  (0.05) | 0.077  (0.05) | 0.475  (0.04) | 0.080  (0.05) | 0.095  (0.05) | 0.476  (0.04) |
