# Supplementary material for: Effectiveness of Shrinkage and Variable Selection Methods for the Prediction of Complex Human Traits using Data from Distantly Related Individuals
Source: Ann Hum Genet. 2015 Jan 20;79(2):122–35. doi: 10.1111/ahg.12099 (PMC4428155; doi:10.1111/ahg.12099)
Supplement: Supplementary file 8 — Table S4 Average (SD, both across 30 replicates) R 2 in validation data sets by simulation scenario, data used for analysis and estimation method. [file AHG-79-122-s008.doc]

**Table S4.** Average (SD, both across 30 replicates) R-squared in validation data sets by simulation scenario, data used for analysis and estimation method.

| Simulation Scenarios . | | | Data Analysis Method & Information Used . | | | | | | | | |
| --- | --- | --- | --- | --- | --- | --- | --- | --- | --- | --- | --- |
| Number of Large Effect QTL | % of Genetic Variance Explained by Large Effect QTL | Sampl-ing of QTL | GBLUP . | | | BayesA . | | | Spike-Slab . | | |
|  |  |  | Markers | Markers+QTL | QTL | Markers | Markers+QTL | QTL | Markers | Markers+QTL | QTL |
| 50 | 25 | UNIF | 0.031  (0.01) | 0.031  (0.01) | 0.216  (0.03) | 0.082  (0.03) | 0.093  (0.03) | 0.264  (0.05) | 0.097  (0.03) | 0.111  (0.03) | 0.264  (0.04) |
|  |  | LOW-MAF | 0.012  (0.01) | 0.012  (0.01) | 0.201  (0.03) | 0.037  (0.05) | 0.058  (0.03) | 0.267  (0.04) | 0.073  (0.03) | 0.094  (0.03) | 0.255  (0.03) |
|  | 75 | UNIF | 0.025  (0.02) | 0.025  (0.02) | 0.197  (0.04) | 0.264  (0.04) | 0.300  (0.04) | 0.366  (0.05) | 0.309  (0.04) | 0.339  (0.04) | 0.373  (0.04) |
|  |  | LOW-MAF | 0.011  (0.01) | 0.010  (0.01) | 0.185  (0.04) | 0.227  (0.04) | 0.277  (0.04) | 0.370  (0.03) | 0.281  (0.04) | 0.327  (0.04) | 0.362  (0.03) |
| 250 | 25 | UNIF | 0.027  (0.01) | 0.026  (0.01) | 0.212  (0.04) | 0.304  (0.02) | 0.039  (0.02) | 0.244  (0.04) | 0.046  (0.02) | 0.054  (0.02) | 0.240  (0.04) |
|  |  | LOW-MAF | 0.009  (0.01) | 0.009  (0.01) | 0.185  (0.04) | 0.014  (0.01) | 0.014  (0.02) | 0.235  (0.05) | 0.027  (0.02) | 0.033  (0.02) | 0.217  (0.04) |
|  | 75 | UNIF | 0.025  (0.01) | 0.026  (0.01) | 0.189  (0.04) | 0.111  (0.04) | 0.144  (0.04) | 0.304  (0.04) | 0.198  (0.04) | 0.235  (0.05) | 0.321  (0.05) |
|  |  | LOW-MAF | 0.016  (0.01) | 0.016  (0.01) | 0.197  (0.03) | 0.086  (0.03) | 0.108  (0.02) | 0.321  (0.04) | 0.180  (0.05) | 0.224  (0.04) | 0.321  (0.04) |
| None | --- | UNIF | 0.026  (0.02) | 0.025  (0.02) | 0.199  (0.04) | 0.013  (0.04) | 0.028  (0.02) | 0.200  (0.04) | 0.028  (0.02) | 0.034  (0.02) | 0.216  (0.05) |
|  |  | LOW-MAF | 0.011  (0.01) | 0.011  (0.01) | 0.203  (0.03) | 0.005  (0.01) | 0.007  (0.01) | 0.227  (0.03) | 0.008  (0.01) | 0.010  (0.01) | 0.229  (0.03) |
