# Supplementary material for: Effectiveness of Shrinkage and Variable Selection Methods for the Prediction of Complex Human Traits using Data from Distantly Related Individuals
Source: Ann Hum Genet. 2015 Jan 20;79(2):122–35. doi: 10.1111/ahg.12099 (PMC4428155; doi:10.1111/ahg.12099)
Supplement: Supplementary file 9 — Table S5 Correlation and R 2 between human height and genomic predictions in testing data sets by method and testing set. [file AHG-79-122-s009.doc]

**TableS5. Correlation and R-squared between human height and genomic predictions in testing data sets by method and testing set.**

|  | Correlation | | | R-squared | | |
| --- | --- | --- | --- | --- | --- | --- |
| Method | Bayes A | Spike-Slab | GBLUP | Bayes A | Spike-Slab | GBLUP |
| run 1 | 0.238 | 0.244 | 0.247 | 0.068 | 0.065 | 0.067 |
| run 2 | 0.107 | 0.106 | 0.109 | 0.001 | 0.003 | 0.007 |
| run 3 | 0.122 | 0.133 | 0.130 | 0.003 | 0.014 | 0.012 |
| run 4 | 0.153 | 0.155 | 0.180 | 0.021 | 0.025 | 0.034 |
| run 5 | 0.138 | 0.146 | 0.148 | 0.016 | 0.022 | 0.023 |
| run 6 | 0.254 | 0.269 | 0.261 | 0.061 | 0.058 | 0.057 |
| run 7 | 0.231 | 0.228 | 0.233 | 0.053 | 0.050 | 0.052 |
| run 8 | 0.131 | 0.137 | 0.146 | 0.011 | 0.019 | 0.021 |
| run 9 | 0.142 | 0.152 | 0.166 | 0.012 | 0.021 | 0.027 |
| run 10 | 0.205 | 0.232 | 0.219 | 0.045 | 0.053 | 0.049 |
| run 11 | 0.170 | 0.176 | 0.194 | 0.031 | 0.035 | 0.041 |
| run 12 | 0.157 | 0.158 | 0.160 | 0.029 | 0.033 | 0.034 |
| run 13 | 0.117 | 0.146 | 0.115 | 0.004 | 0.020 | 0.007 |
| run 14 | 0.128 | 0.126 | 0.133 | 0.010 | 0.014 | 0.016 |
| run 15 | 0.174 | 0.153 | 0.178 | 0.035 | 0.029 | 0.038 |
| run 16 | 0.143 | 0.155 | 0.164 | 0.024 | 0.033 | 0.036 |
| run 17 | 0.210 | 0.221 | 0.227 | 0.044 | 0.048 | 0.052 |
| run 18 | 0.199 | 0.214 | 0.217 | 0.040 | 0.045 | 0.047 |
| run 19 | 0.176 | 0.178 | 0.200 | 0.034 | 0.035 | 0.043 |
| run 20 | 0.083 | 0.103 | 0.109 | -0.004 | 0.010 | 0.011 |
| run 21 | 0.089 | 0.096 | 0.105 | -0.011 | 0.000 | 0.002 |
| run 22 | 0.126 | 0.128 | 0.141 | 0.005 | 0.012 | 0.016 |
| run 23 | 0.171 | 0.175 | 0.185 | 0.030 | 0.034 | 0.037 |
| run 24 | 0.209 | 0.195 | 0.204 | 0.043 | 0.037 | 0.041 |
| run 25 | 0.124 | 0.129 | 0.122 | 0.026 | 0.033 | 0.030 |
| run 26 | 0.120 | 0.136 | 0.145 | 0.010 | 0.020 | 0.023 |
| run 27 | 0.134 | 0.139 | 0.137 | 0.014 | 0.021 | 0.019 |
| run 28 | 0.160 | 0.166 | 0.160 | 0.021 | 0.027 | 0.025 |
| run 29 | 0.187 | 0.181 | 0.179 | 0.034 | 0.033 | 0.032 |
| run 30 | 0.174 | 0.172 | 0.163 | 0.033 | 0.034 | 0.031 |
| average | 0.159 | 0.165 | 0.169 | 0.025 | 0.029 | 0.031 |
| sd | 0.044 | 0.043 | 0.043 | 0.019 | 0.016 | 0.016 |
